# Plantar Loading Reflects Ulceration Risks of Diabetic Foot with Toe Deformation

**DOI:** 10.1155/2015/326493

**Published:** 2015-03-16

**Authors:** Y. C. Lu, Q. C. Mei, Y. D. Gu

**Affiliations:** ^1^Zhejiang Wanli University, No. 8, South Qian Hu Road, Ningbo, Zhejiang 315100, China; ^2^Faculty of Sports Science, Ningbo University, No. 818, Fenghua Road, Ningbo 315211, China

## Abstract

Diabetes has been one of the most common chronic diseases all over the world. The purpose of this study was to quantitatively assess the foot loading characteristics of diabetic patients with fifth-toe deformity through a comparative analysis with diabetic patients with healthy and normal feet. Six neuropathic diabetic female subjects with the fifth-toe deformation and six age-matched neuropathic diabetic controls without any feet deformities participated in the walking test. Dynamic barefoot plantar pressure was measured with Novel EMED force plate. Peak pressure and pressure-time integral for all 7 foot regions (rearfoot, midfoot, lateral forefoot, central forefoot, medial forefoot, great toe, and other toes) were collected. Peak pressure was significantly higher in the patients with toe deformity in rearfoot, central forefoot, and great toe regions compared with the control group. Meanwhile, loading sustaining period extended longer in great toe region of deformed group than in that of the control group, and the center of pressure was nearly in the big toe region during toe offstage. Diabetic patients with fifth-toe deformity could have plantar contact area reduction in the other toes part and increased loading to the great toe part. The result showed that fifth-toe deformity was associated with potential ulceration risk especially in hallux region.

## 1. Introduction

Diabetes mellitus has been one of the most common chronic diseases that influenced people's normal daily life. It has been estimated that the number of people with diabetes all over the world will surpass 365 million by 2030 [1]. Diabetic foot with foot ulceration or foot deformation hinders the normal locomotion [2]. It was the result of long-term loading to the foot plantar surface during gait that changed or transferred in particular regions among patients with diabetes mellitus and peripheral neuropathy [2–4]. Some excessive pressures in feet that lack protective sensation are considered as a major risk factor for plantar ulceration [3], which is the most common precursor to lower extremity amputation among patients with diabetes [5]. Peripheral neuropathy could arouse the emerging of lower limb injuries and even disability among the diabetes patients. Due to the decreasing foot nerve sensory, the foot injury could easily be overlooked, thus increasing the risk of skin ulcerations or damage [3, 4, 6]. At least 15% of these ulcerations would lead to some forms of foot amputation. Previous study found that early diabetic foot tends to be toes deformities [7–9] and imbalance in the muscles to the foot or lower extremity [10], then causing abnormal plantar pressure [11]. When the lesion or dysfunction of the human foot structure and human locomotion ability deteriorated, plantar pressure and foot loading distribution would change consequently [12, 13].

It was clear that the emergence of the diabetic foot ulceration, to a great extent, was caused by the changes of foot loading characteristics [14]. And diabetic foot could change the plantar pressure distribution, causing the blood flow of the foot being unevenly distributed, thereby destroying the blood supply to the foot, finally leading to foot ulceration and even amputation [13]. However, there was rare report about kinetic changes from toes deformation, which was an early sign of diabetic foot deformation and the major reason for the change of plantar loading [15]. Therefore, this study was aimed at measuring the plantar pressure of diabetes patients of fifth-toe deformation. The analysis of the plantar pressure distribution during normal walking was conducted and the foot loading characteristics of feet with fifth-toe deformity were illustrated with comparison with diabetes patients of healthy and normal feet, intending to supply some useful suggestions for diabetic shoes designing, alleviating the pain in patients' deformed feet, and reducing the incidence of diabetic foot ulceration or even amputation.

## 2. Materials and Methods

### 2.1. Participants

**Figure 1:**
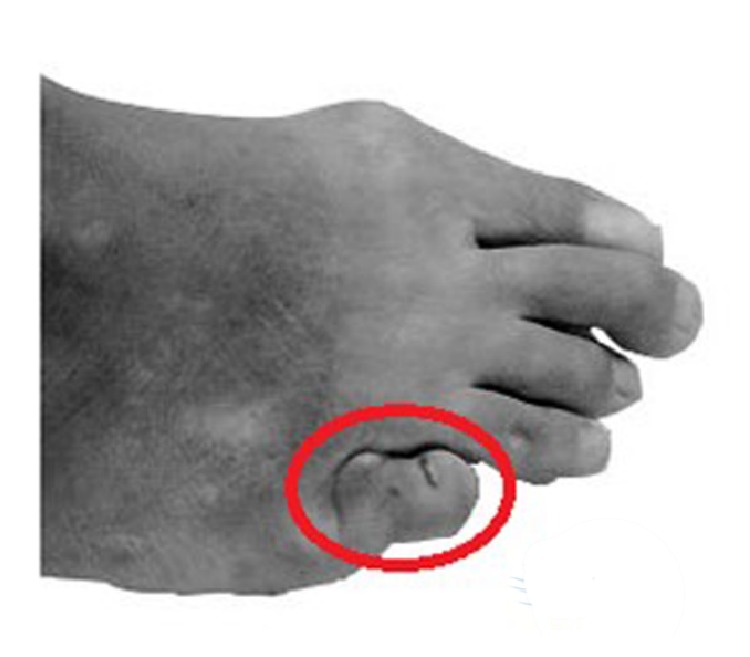
Illustration of the fifth-toe deformation.

A total of twelve age-matched diabetic female participants joined in the experiment, six of them with toes lesions and another six with healthy feet. Subjects in the experimental group were elected based on deformity presented in the fifth toe under non-weight-bearing condition (Figure 1), considering the hammered toes or valgus hallux leading to deformities not likely to display significant biomechanical differences [16, 17]. All of them had no history of surgeries or any rehabilitation treatment to their feet. Although the deformed fifth toe was of claw or hammer shape, but with no sign of festering to feet, both deformed and control group participants could walk normally. This study was approved by the Ethics Committee of Ningbo University. Before the experiment, the subjects were informed of requirements and procedures of the walking test. All gave informed written consent to participate in the study. The basic demographic information was shown in Table 1.

### 2.2. Experiment Protocols and Statistical Analysis

The Novel EMED pressure measuring plate (Novel GmbH, Munich, Germany) was used for plantar pressure data measurement. Before the experiment, participants were required to adjust their walking step with right foot landing on the plantar pressure measuring plate, which was fixed in the center of the walkway aisle. During the test, subjects walked along the aisle straightly with their selected and comfortable speed so as to perform their normal gait characteristics. Each participant walked six trials successively to show their normal gait. To accurately and deeply illustrate the plantar loading features, the foot was divided into seven anatomical regions, which are rearfoot part (RF), midfoot part (MF), lateral forefoot part (LFF), central forefoot part (CFF), medial forefoot part (MFF), great toe part (GT), and other toes part (OT). For each region, peak pressure, pressure-time integral, and trajectory of the center of pressure (CoP) were collected and the average of the six walking trials was used for data analysis to minimize error.

All statistical tests were performed using SPSS (version 17.0.) and statistical test data of the two groups of subjects, which are indicators mean, standard deviation, and significant statistics. A significance level of *P* = 0.05 was used for all analyses.

## 3. Results

**Figure 2:**
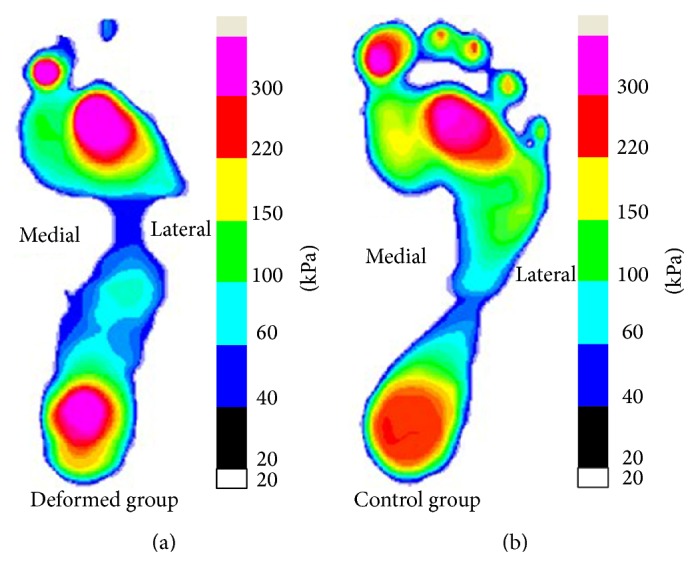
Plantar pressure distribution between the fifth-toe deformed group (a) and control group (b) (∗significant difference, *P* < 0.05).

The plantar pressure distribution patterns showed significant difference (Figure 2) between the fifth-toe deformed group and control group. By comparative analysis of the plantar pressure distribution with quantitative parameters (peak pressure and pressure-time integral) in seven anatomy-related part divisions, significant difference exists between the fifth-toe deformed group (Figure 1) and the control group.

**Figure 3:**
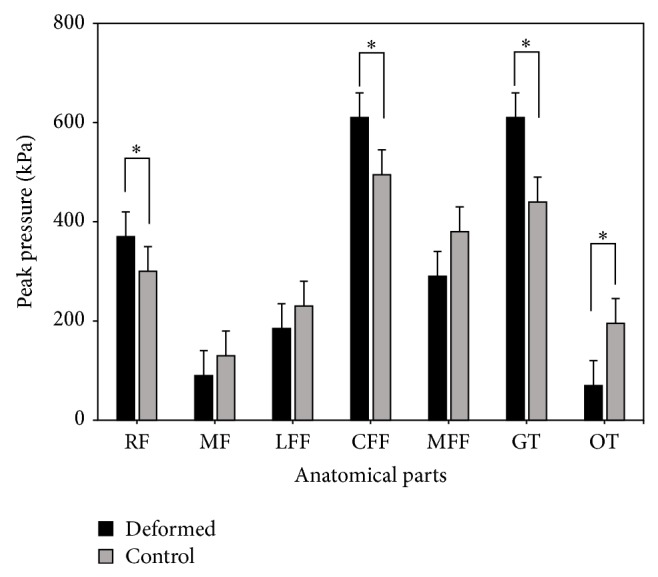
The comparison of peak pressure between the fifth-toe deformed group and control group (∗significant difference, *P* < 0.05).

As to the peak pressure of the deformed group (Figure 3) comparing with the control group, rearfoot (RF), central forefoot (CFF), great toe (GT), and other toes (OT) regions showed significant difference. In the toes region, particularly the other toes (OT) part of the deformed group showed no contact or plantar pressure in the relevant anatomy part compared with the control group (Figure 2). Combining the results of quantitative analysis, the peak pressure of the deformed group was basically focused in the great toe (GT) region and significantly higher than that in the control group. Conversely, the peak pressure in the OT region was obviously smaller than that of control group. The OT region, particularly the fifth toe, showed no contact with the force plate while comparing with the control group showing obvious pressure in the 2–5 toes area. When comes to the peak pressure in the forefoot part, the central forefoot (CFF) of fifth-toe deformed group showed significantly higher peak pressure than that of control group, differently, lateral forefoot (LFF) and medial forefoot (MFF) areas showed no significance comparing with the control group. The peak pressure of RF showed significance, with deformed group higher than control group.

To sum up these characteristics, the peak pressure of the deformed group, central forefoot (CFF), and the great toe (GT) areas was relatively concentrated. It should be noted that, in other toes (OT) area, the peak pressure of the deformed group was also significantly less than the control group; the pressure value of the other toes region only took up one-third the value shown in the control group while the peak pressure value in GT was 32% higher than that appeared in the control group.

**Figure 4:**
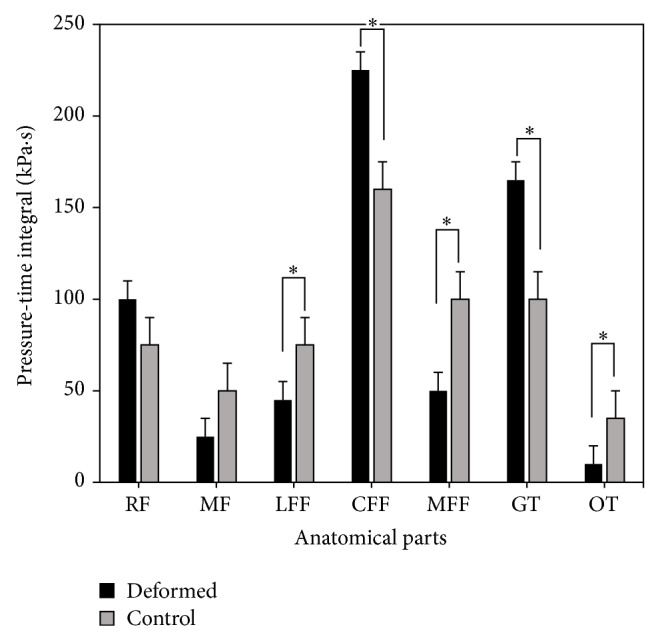
Pressure-time integral comparison between the fifth-toe deformed group and control group (∗significant difference, *P* < 0.05).

Comparing the pressure-time integrals, the lateral forefoot (LFF), central forefoot (CFF), medial forefoot (MFF), great toe (GT), and the other toes (OT) parts showed significant difference. But as to the pressure-time integral in the GT and CFF regions, deformed group were significantly larger than those of the control group (Figure 4). The result compared with peak pressure features showed different patterns, mainly in the RF, LFF, and MFF areas. For RF region, the pressure-time integral did not show significant difference, but in the LFF and MMF regions, no significance was illustrated in peak pressure value.

**Figure 5:**
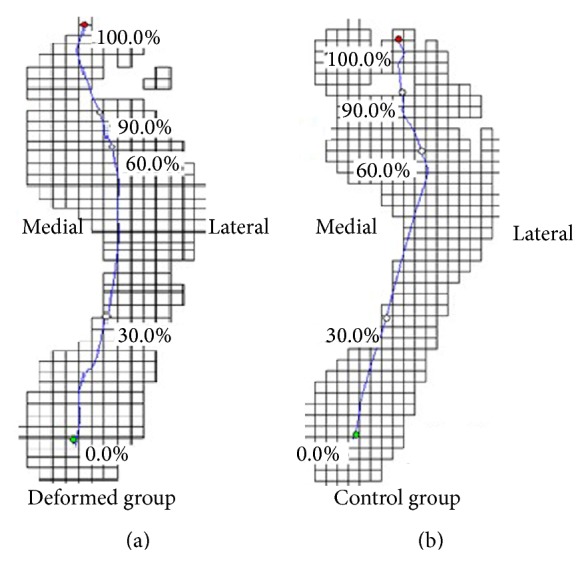
The center of pressure (CoP) trajectory of fifth-toe deformed group (a) and control group (b) during walking.

For the trajectory of center of pressure (CoP) (Figure 5), the deformed group and the control group did not show significant difference of the CoP point in the rearfoot (RF) and midfoot (MF) regions but showed significant differences in the forefoot (MFF, CFF, and LFF) and toe (GT and OT) areas. Particularly, for the CoP line in the forefoot area, the deformed group showed slight tendency of medial shift, while the control group offset amplitude increases. The present findings confirm recent reports from forefoot plantar pressure group with lesions mainly in the middle of the forefoot, and the pressure of the control group is consistently concentrated in central and medial forefoot regions. From the stance period of the total pressure out of the center line of the forefoot area, it can be easily concluded that the proportion time of the deformed group was significantly less than that of the control group, while, in the toe area, the percentage of the deformed group's time was significantly greater than that of the control group. We need to stress that, in the toe region, the pressure center of the deformed group completely shifted to the hallux region, while the control group remained between the first and second toes.

## 4. Discussion

The clinical importance of studying the biomechanics of toe deformity in neuropathic diabetic patients has been demonstrated by several prospective population-based studies which showed that this structure abnormality was a significantly independent predictor of plantar ulceration in diabetic patients [14, 15]. The results in this study clearly showed that the fifth-toe deformity was associated with significantly changed foot loading parameters, such as peak pressure, pressure-time integral, and center of pressure (CoP) trajectory at the plantar surface. These findings reflected that the diabetic foot with fifth-toe deformation caused more impact loading during the patient's walking gait.

From the plantar pressure point of view, the CoP line of the deformed group transfers to the hallux part during walking. Because the fifth toe has been significantly deformed that was unable to withstand greater pressure, it showed obvious reduction of peak pressure in the lateral forefoot and other toes regions. The contact area in the other toes (OT) region of the deformed group is significantly less that of the control group, which is similar with the previous studies [16]. One important function of the toes in gait was to contact the surface and exert sufficient pressure to obtain a fixed point from which the body can be propelled [18]. Owing to the toe deformation and reduced contact area, the toes plantar pressure will shift to the forefoot part, which originally alleviates the impact to the metatarsal region in the pushing-off phase. The fifth-toe deformed patients showed that their plantar contact area decreased and the pressure-time integral increased significantly. Unlike other deformations, reduced toe functions may be compensated with increased loading to other areas. When toes' function declines, the pressure that the forefoot (metatarsal part) withstands in the pushing-off phase will significantly increase and the loading that toes bearing would decrease. The results of these findings were inconsistent with the finding of earlier studies [19], and toes withstanding collision force area and pressure were significantly reduced in the diabetic patients. This would cause the pressure transfer to the nearby area with the pressure peak increasing [20]. However, the peak pressure expansion will cause necrosis of the muscle tissue and muscle fiber atrophy and exacerbates the risk of lesion in these pressure peak increased areas [21–23]. The reason for peak pressure increasing in the fifth-toe deformed group may be due to toe deformity leading to fat tissue destruction, leading to no capacity to bear tremendous pressure, so that the pressure in metatarsal region elevated in order to maintain the body stabilization [22].

Compared with the control group, the pressure-time integral of toe deformed group showed significant reduction in lateral and medial forefoot and other toes regions, while great toe demonstrated significantly greater one than that of the control group [9]. Combining stage centers of pressure line percentage in the gait cycle, the time of pressure center used in forefoot was significantly less than that of the control group and in the toe region it was significantly greater than that of the control group when walking. There were some studies reporting that diabetic patients' plantar pressure time was significantly longer than normal, which may be another reason that diabetes patients are prone to plantar pressure abnormalities [24]. The present findings confirmed recent reports from which it was found that the pressure-time integral of the lesion area in toe deformed diabetic patients was significantly less than that of control group, in comparison with the pressure centerline chart (Figure 5), which may be an important factor contributing to lesions. Pressure-time integral of the deformed group was significantly smaller than the control group in OT region, which showed that the deformed group had been rarely used in this area to withstand the pressure to reduce the impact to the region. The present findings confirm recent reports from which it was found that the pressure-time integral of the lesion area was significantly less than that of the control group with full bottom pressure distribution of hallux lesion diabetes [16, 23]. This situation may cause the peak pressure transfer to the neighboring region and result in the peak pressure being assigned to the wrong region [6]. The pressure of the deformed group was mainly concentrated in the middle forefoot and the hallux regions. As to pressure to other toes (OT) region, the pressure loading was basically withstood by the hallux and OT region was exposed with less loading. Conversely, the pressure loading of the control group in toes (Hallux and Other Toes) part was shared by the great toe and other toes. This would be directly linked with the locational ulcerations risks, though subjects in this study not yet showing any sign of ulcers. Considering these factors, functional insoles or shoes could accordingly be adjusted and utilized to alleviate the pressure or loading to those areas, which might be of greatly preventive or even rehabilitation effect for diabetic ulcerations [25–27].

One limitation of this study was that the kinematic analysis of diabetic deformed feet were not taken to investigate the lower extremity motion characters, particularly the ankle movement in the sagittal plane (range of motion, ROM). This should be the next step to further reveal the gait patterns of fifth-toe deformed diabetic patients.

## 5. Conclusion

This study found that the great toe and central forefoot regions are the foot loads bearing parts in the fifth-toe deformity group and the most sensitive areas to foot ulceration due to long-term collapse impact. The changes of plantar pressure distribution and center of pressure trajectory should be considered while analyzing and treating diabetic-deformation-related plantar ulcers. The quantitative illustration of foot loading characteristics of fifth-toe deformity could be of great beneficial for diabetic patients to understand their foot loading conditions and doctors or rehabilitation therapist to make protocols to prevent or even rehabilitate fifth-toe-deformation-related foot ulceration and other traumas. Some functional footwear is necessary to use so as to reduce the risks of ulceration in relevant regions, normalizing the distribution of diabetic patients with fifth-toe deformity.

## Figures and Tables

**Table 1:** Participants' basic demographic information.

	Disease group		Control group		*P* value
	Average	SD	Average	SD	
Age (years)	50.2	11.73	44.5	6.63	0.341
Height (cm)	155.7	4.32	158.3	4.23	0.305
Weight (kg)	55.5	7.84	61.3	8.29	0.239
Diabetes duration (years)	8.6	2.28	7.2	1.94	0.251
Body mass index (kg/m^2^)	22.9	2.84	24.4	2.73	0.354
